# Visual Cortical Entrainment to Motion and Categorical Speech Features during Silent Lipreading

**DOI:** 10.3389/fnhum.2016.00679

**Published:** 2017-01-11

**Authors:** Aisling E. O’Sullivan, Michael J. Crosse, Giovanni M. Di Liberto, Edmund C. Lalor

**Affiliations:** ^1^School of Engineering, Trinity College DublinDublin, Ireland; ^2^Trinity Centre for Bioengineering, Trinity College DublinDublin, Ireland; ^3^Department of Pediatrics and Department of Neuroscience, Albert Einstein College of MedicineBronx, NY, USA; ^4^Trinity College Institute of Neuroscience, Trinity College DublinDublin, Ireland; ^5^Department of Biomedical Engineering and Department of Neuroscience, University of RochesterRochester, NY, USA

**Keywords:** EEG, visual speech, lipreading/speechreading, visemes, motion, temporal response function (TRF), EEG prediction

## Abstract

Speech is a multisensory percept, comprising an auditory and visual component. While the content and processing pathways of audio speech have been well characterized, the visual component is less well understood. In this work, we expand current methodologies using system identification to introduce a framework that facilitates the study of visual speech in its natural, continuous form. Specifically, we use models based on the unheard acoustic envelope (E), the motion signal (M) and categorical visual speech features (V) to predict EEG activity during silent lipreading. Our results show that each of these models performs similarly at predicting EEG in visual regions and that respective combinations of the individual models (EV, MV, EM and EMV) provide an improved prediction of the neural activity over their constituent models. In comparing these different combinations, we find that the model incorporating all three types of features (EMV) outperforms the individual models, as well as both the EV and MV models, while it performs similarly to the EM model. Importantly, EM does not outperform EV and MV, which, considering the higher dimensionality of the V model, suggests that more data is needed to clarify this finding. Nevertheless, the performance of EMV, and comparisons of the subject performances for the three individual models, provides further evidence to suggest that visual regions are involved in both low-level processing of stimulus dynamics and categorical speech perception. This framework may prove useful for investigating modality-specific processing of visual speech under naturalistic conditions.

## Introduction

It is well established that during face-to-face conversation visual speech cues play a prominent role in speech perception and comprehension (Summerfield, 1992; Campbell, 2008; Peelle and Sommers, 2015). It has been shown that audiovisual (AV) speech processing benefits from the visual modality at several hierarchical levels of linguistic unit, including syllables (Bernstein et al., 2004), words (Sumby and Pollack, 1954) and sentences (Grant and Seitz, 2000), and that this gain is present in both noisy (Sumby and Pollack, 1954; Ross et al., 2007; Crosse et al., 2016b) and noise-free conditions (Reisberg et al., 1987; Crosse et al., 2015a). Research on the anatomical organization of auditory speech processing has established a pathway of hierarchical processing, where each level encodes acoustic features of different complexity (Hickok and Poeppel, 2007). And while several studies have reported auditory cortical activation to silent lipreading (Sams et al., 1991; Calvert et al., 1997; Pekkola et al., 2005), the role of this activation remains unclear, i.e., whether it simply serves a modulatory function (Kayser et al., 2008; Falchier et al., 2010) or actually categorizes visual speech features. If the latter were true, one would expect auditory cortical activity during silent speech to track the visual speech features, yet there is a lack of strong evidence of sustained tracking by auditory regions to continuous visual speech (Crosse et al., 2015b). This, coupled with reports of activation of high-level visual pathways during speech reading, has fueled the theory that visual cortex may be capable of processing and interpreting visual speech (for review see Bernstein and Liebenthal, 2014).

Several recent studies have sought to further characterize the role of visual cortex in speech perception. Using cortical surface recordings, stronger visual cortical activity has been observed in response to silent word onsets than AV words (Schepers et al., 2015). This may represent visual cortex accessing word meaning in the absence of an informative audio input. And fMRI research on AV speech perception has shown increases in connectivity strength between putatively multisensory regions and visual cortex when the visual modality is more reliable (Nath and Beauchamp, 2011). In terms of continuous visual speech processing, recent MEG work found extensive (bilateral) entrainment of visual cortex to visual speech (lip movements) when the visual signal was relevant for speech comprehension (Park et al., 2016). Importantly, this entrainment was restricted to a much smaller area in early visual cortex (left-lateralized) when the visual speech was irrelevant. Another study that reconstructed an estimate of the acoustic envelope from occipital EEG data recorded during silent lipreading found a strong correlation between reconstruction accuracy and lipreading ability, suggesting that visual cortex encodes high-level visual speech features (Crosse et al., 2015a). This is supported by behavioral research which has shown that visually presented syllables are categorically perceived (Weinholtz and Dias, 2016).

Electrophysiological evidence of categorical processing in the context of natural visual speech is lacking. Part of the reason for this is that researchers have focused on studying brain responses to discrete visual syllables, audio-speech envelope entrainment measures, and responses to lip and facial movements. Although studying how the brain encodes features such as the speech envelope and lip movements can inform our understanding of visual speech processing, such simplified speech parameters overlook higher-level categorical processing which may be present. Efforts to further parameterize visual speech have involved the application of multiple sensors to the face and tongue of the speaker (Jiang et al., 2002; Bernstein et al., 2011), a method that is time consuming and yet still cannot fully capture the diverse array of complex motion involved in the production of speech. Here, we take a simplified approach to quantifying visual speech by characterizing the low-level temporal information in the form of the acoustic envelope (given its correlation with speech movements, Chandrasekaran et al., 2009) and the frame-to-frame motion signal, as well as the higher-level linguistic information as groupings of visually similar phonemes, i.e., visemes (Fisher, 1968). A system identification technique is employed to map these features to the subject’s EEG by calculating the so-called temporal response function (TRF) of the system (Crosse et al., 2016a). These TRFs are then tested in their ability to predict unseen EEG data using Pearson’s correlation (*r*). The variation in these EEG prediction accuracies across different models is used as a dependent measure for assessing how well the EEG reflects the processing of lower- and higher-level visual speech features. The overarching hypothesis is that visual cortex encodes both the low-level, motion-related features of visual speech, as well as the higher-level, categorical articulatory features. In testing this hypothesis, we aim to establish a framework that facilitates the study of natural visual speech processing in line with methods previously used to characterize the hierarchical organization of speech in the auditory modality (Lalor and Foxe, 2010; Di Liberto et al., 2015).

## Materials and Methods

The EEG data analyzed here were collected as part of a previous study. A more detailed account of the participants, stimuli and experimental procedure can be found in Crosse et al. (2015a).

### Subjects

Twenty-one native English speakers (8 females; age range: 19–37 years), none of which were trained lipreaders, gave written informed consent. All participants were right-handed, free of neurological diseases, had self-reported normal hearing and normal or corrected-to-normal vision. This study was carried out in accordance with the Declaration of Helsinki. The protocol was approved by the Ethics Committee of the Health Sciences Faculty at Trinity College Dublin, Ireland.

### Stimuli and Procedure

**Figure 1:**
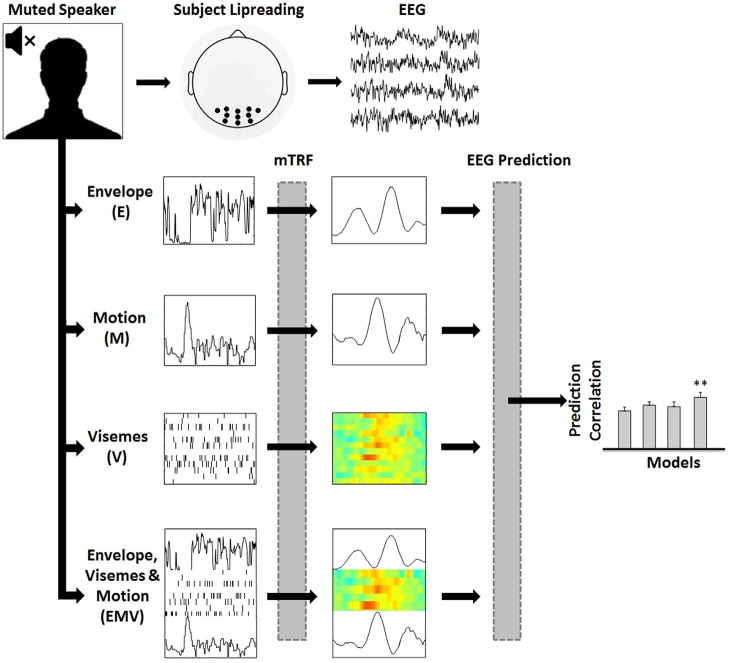
**Assessing the representation of visual speech in EEG (adapted from Di Liberto et al., 2015).** 128-channel EEG data were recorded while subjects watched videos of continuous, natural speech consisting of a well-known male speaker. Linear regression was used to fit multivariate temporal response functions (mTRFs) between the low-frequency (0.3–15 Hz) EEG recordings and seven different representations of the speech stimulus (EV, MV and EM models are not shown). Each mTRF model was then tested for its ability to predict EEG using leave-one-out cross-validation.

The speech stimuli were drawn from a collection of videos featuring a well-known male speaker. The videos consisted of the speaker’s head, shoulders and chest, centered in the frame (see Figure 1). The speech was conversational like, and the linguistic content focused on political policy. Stimulus presentation and data recording took place in a dark, sound-attenuated room with participants seated at a distance of 70 cm from the visual display. Visual stimuli were presented on a 19″ CRT monitor operating at a refresh rate of 60 Hz. Fifteen 60-s videos were rendered into 1280 × 720-pixel movies in VideoPad Video Editor (NCH Software). Soundtracks were deleted from the 15 videos which had a frame rate of 30 frames per second. Participants were instructed to fixate on the speaker’s mouth while minimizing eye blinking and all other motor activity during recording. The study from which the data are taken involved seven conditions, most of which included audio speech (Crosse et al., 2015a). Each of the 15 videos were presented seven times to each subject, once per condition. Presentation order was randomized across conditions and videos. This work examines EEG recordings from the visual-only condition.

To encourage active engagement with the video content, participants were required to respond to target words via button press. Before each trial, a target word was displayed on the monitor until the participant was ready to begin. A target word could occur between one and three times in a given 60-s trial. This allowed identification of whether subjects were successful at lipreading or not. A different set of target words was used for each condition to avoid familiarity, and assignment of target words to the seven conditions was counterbalanced across participants. This, combined with the randomized presentation order of the 15 videos, made it quite unlikely that subjects would be able to recognize the silent video from a previously heard audio/AV version.

### Visual Speech Representations

To investigate mappings between different representations of visual speech and low-frequency (0.3–15 Hz) EEG, we defined seven representations of visual speech (Figure 1): (1) the broadband amplitude envelope of the corresponding acoustic signal (E); (2) the frame-to-frame motion of the video (M); (3) a time aligned sequence of viseme occurrences (V), and (4–7) respective combinations of each of the individual models, i.e., EV, MV, EM and EMV.

Previous work has shown that the motion of the mouth during speech is correlated with the acoustic speech envelope (Chandrasekaran et al., 2009). Therefore, the speech envelope can be thought of as a proxy measure of the local motion related to the mouth area, even though the subjects were not actually presented with the acoustic speech. The broadband amplitude envelope representation was obtained by bandpass filtering the speech signal into 256 logarithmically-spaced frequency bands between 80 Hz and 3000 Hz using a gammachirp filterbank (Irino and Patterson, 2006). The envelope at each of the 256 frequency bands was calculated using a Hilbert transform, and the broadband envelope was obtained by averaging over the 256 narrowband envelopes.

To more explicitly represent the motion in the videos, we calculated their frame-to-frame motion. For each frame, a matrix of motion vectors was calculated using an “Adaptive Rood Pattern Search” block matching algorithm (Barjatya, 2004). A measure of global motion flow was obtained by calculating the sum of all motion vector lengths of each frame (Bartels et al., 2008). This was then upsampled from 30 Hz to 128 Hz to match the rate of the EEG data.

Previous work involving visual speech identification tasks have demonstrated groupings of phonemes which, when presented visually were perceptually similar (consider that a /p/ and a /b/ cannot be distinguished by vision alone;Woodward and Barber, 1960; Fisher, 1968). Each class can thus be defined as the smallest perceptual unit of visual speech, i.e., a viseme. To derive a viseme representation from our videos, we first obtained a phonemic representation as in Di Liberto et al. (2015), and then converted that to visemes based on the mapping defined in Auer and Bernstein (1997). Combined models (e.g., EMV) were formed by concatenating the individual models stimuli, resulting in a model whose dimension is equal to the sum of the dimension of each individual model. The phoneme-to-viseme transformation means that timing of our viseme representation is actually tied to the acoustic boundaries rather than the visual. Since features of visual speech have a complex temporal relationship with the sound produced (Chandrasekaran et al., 2009; Schwartz and Savariaux, 2014), the time window that provided a qualitatively good alignment with the other models (E and M) was 150 ms earlier for the viseme model. As will become clear below, taking account of this fact also enabled us to use a consistent time window across all individual models and combined models in relating the speech stimulus to the EEG, thereby ensuring that our comparison across models was fair.

### EEG Acquisition and Pre-Processing

Continuous EEG data were acquired using an ActiveTwo system (BioSemi) from 128 scalp electrodes. The data were low-pass filtered online below 134 Hz and digitized at a rate of 512 Hz. Triggers were sent by an Arduino Uno microcontroller which detected an audio click at the start of each soundtrack to indicate the start of each trial. Subsequent pre-processing was conducted offline in MATLAB; the data were bandpass filtered between 0.3 Hz and 15 Hz, then downsampled to 128 Hz and re-referenced to the average of all channels. To identify channels with excessive noise, the time series were visually inspected in Cartool (Brunet, 1996), and the standard deviation of each channel was compared with that of the surrounding channels in MATLAB. Channels contaminated by noise were replaced by spline-interpolating the remaining clean channels with weightings based on their relative scalp location in EEGLAB (Delorme and Makeig, 2004).

### Temporal Response Function Estimation

In order to relate the continuous EEG to the various visual speech representations introduced above, we use a regression analysis that describes a mapping from one to the other. This mapping is known as a TRF and was computed using a custom-built toolbox in MATLAB (Crosse et al., 2016a). A TRF can be thought of as a filter that describes how a particular stimulus feature (e.g., the acoustic envelope) is transformed into the continuous EEG at each channel. So if *s*(*t*) represents the stimulus feature at time *t*, the EEG response at channel *n*, *r*(*t*, *n*), can be modeled as a convolution with a to-be-estimated TRF, *w*(τ, *n*).

(1) $$r(t,n)\;=\;\sum_{\tau \;=\;T_{\text{min}}}^{T_{\text{max}}}w(\tau ,n)s(t-\tau )+\varepsilon (t,n),$$

where *ε*(*t*, *n*) is the residual response at each channel not explained by the model. Of course, the effect of a stimulus event is not seen in the EEG until several tens of milliseconds later and lasts for several hundred milliseconds. So, the TRF is defined across a certain set of time-lags between stimulus and response (*T*_min_ − *T*_max_). In our case, we fit TRFs for each 60-s trial using ridge regression expressed in the following matrix form:

(2) $$w\;=\;(S^{T}S\;+\;\lambda I{)}^{-1}\;S^{T}r,$$

where λ is the ridge parameter, chosen to optimize the stimulus-response mapping, *S* is a matrix containing a time series of stimulus samples for the window of interest (i.e., the lagged time series), *r* is a matrix of all 128 channels of neural response data, and *I* is the identity matrix which provides regularization and prevents overfitting. For a more detailed explanation of this approach, see Crosse et al. (2016a).

### EEG Prediction and Model Evaluation

We wished to use this TRF modeling approach to assess how well each of the abovementioned visual speech features was being encoded by visual cortex. To do this, we fit TRFs describing the mapping between each feature and the EEG. Then, using leave-one-out cross-validation, we assess how well we could predict unseen EEG data using the different models. If one can predict EEG with accuracy greater than chance using a particular model or combination of models, one can assert with some confidence that the EEG is reflecting the encoding of that particular feature or set of features. Because we had 15 trials for each subject, leave-one-out cross-validation meant that each TRF was fit to the data from 14 trials and then the average TRF across these 14 trials was used to predict the EEG in the remaining trial (Crosse et al., 2016a).

Prediction accuracy was measured by calculating Pearson’s (*r*) linear correlation coefficient between the predicted and original EEG responses at each electrode channel. The time window that best captures the stimulus-response mapping is used for EEG prediction (i.e., *T*_min_, *T*_max_). This is identified by examining the TRFs on a broad time window (e.g., −200 ms to 500 ms) and then choosing the temporal region of the TRF that includes all relevant components that map the stimulus to the EEG with no evident response outside of this range (e.g., 30–380 ms for E, M and V models). This time window is also used for the combined models so that differences in performance are not affected by the choice of time window. To optimize performance within each model, we conducted a parameter search (over the range 2^−20^, 2^−16^… 2^20^) for the regularization parameter λ that maximized the correlation between the predicted and recorded EEG. To prevent overfitting, the λ values were chosen as the value corresponding to the highest mean prediction accuracy across the 15 trials for each subject. The cross-validation is then re-run for each model with a constricted range of λ values, based on the range that includes the optimum λ value for each subject. Since the cross-validation procedure takes the average performance across trials, the models are not biased towards the test data used for cross-validation. As a result, the TRF is more generalized and capable of predicting new unseen data with a similar accuracy. This procedure is explained in more detail in Crosse et al. (2016a). After model optimization, a set of 11 electrodes from the occipital region of the scalp (represented by black dots in Figure 1) were selected for calculating EEG prediction accuracy because of their consistently high prediction correlations. A nonparametric test was performed in Cartool to test for topographical differences in prediction accuracies across models (i.e., a T-ANOVA). Importantly, there was no statistical difference in the topographic distribution of these predictions between the models (*p* > 0.05), thus ensuring electrode selection did not bias any of the models.

All statistical analyses were conducted using one-way repeated-measures ANOVAs. *Post hoc* comparisons were conducted using two-tailed paired *t*-tests. The level of chance was obtained by calculating the correlation between the predicted EEG and five randomly selected EEG trials from the remaining fourteen. The averages of these predictions (for all models) were then pooled together and the chance level was taken as the 95th percentile of these values. All numerical values are reported as mean ± SD.

## Results

To identify neural indices of lower- and higher-level speech reading, we investigated the neural response functions that mapped different representations of visual speech to the low-frequency (0.3–15 Hz) EEG from 11 bilateral occipital electrodes (Figure 1) of subjects attending to natural visual speech.

### Envelope, Motion and Visemes are Reflected in EEG

**Figure 2:**
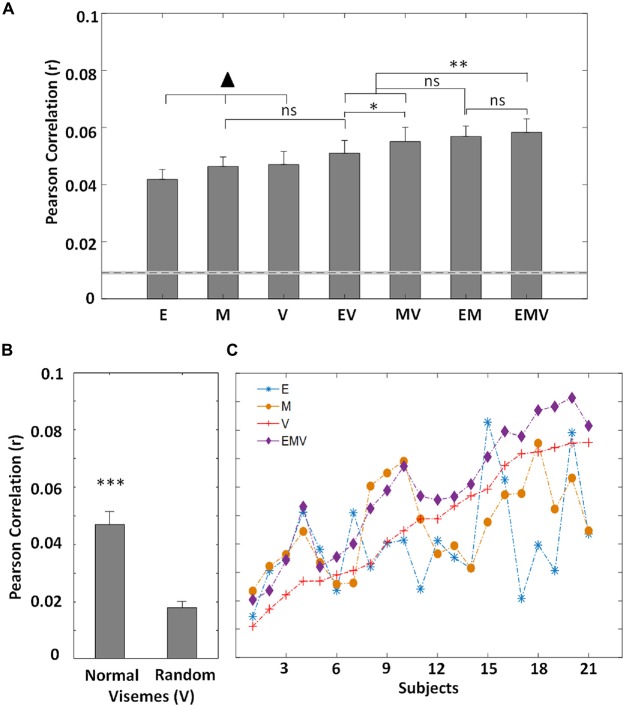
**(A)** Grand-average (*N* = 21) EEG prediction correlations (Pearson’s *r*) for the visual speech models (mean ± SEM) for low-frequency EEG (0.3–15 Hz). The ▴ indicates the models other models (▴ *p* < 0.05), except for EV vs. M (*p* = 0.263). There is no difference in performance between these three models (*p* > 0.05). The dotted line represents the 95th percentile of chance-level prediction accuracy. **(B)** The prediction accuracy (*N* = 21) for normal and randomized visemes within their active time points. **(C)** Correlation values between recorded EEG and that predicted by each mTRF model for individual subjects. The subjects are sorted according to the prediction accuracies of the viseme model. (*p < 0.05, **p < 0.005, ***p < 0.001).

As mentioned above, the acoustic speech envelope can be thought of as a proxy measure for mouth movement (Chandrasekaran et al., 2009), or may reflect the tracking of visual speech features (Crosse et al., 2015a). Thus, the use of the envelope model to predict EEG activity sought to investigate further the nature of its representation in visual cortex. The motion model (M) accounts for both local and global motion present (Bartels et al., 2008) which may be related (e.g., cheek, jaw, eye movements) or unrelated (e.g., movement during pauses) to speech. Thus, this model serves to represent the low-level information received by visual cortex during lipreading. The relationship between low-frequency EEG and a categorical phoneme representation of speech has previously been examined for audio speech (Di Liberto et al., 2015). However, no such relationship has been investigated for visual speech. Transforming phonemes into a lower-dimensional viseme representation (V), by grouping visually indistinguishable phonemes, allows us to explore the processing of these visual speech features using electrophysiology. Using these visual speech representations, we find that individually the envelope, motion and viseme models perform similarly at predicting EEG (E: 0.040 ± 0.017, M: 0.046 ± 0.015, V: 0.047 ± 0.021; *F*_(2,40)_ = 1.42, *p* = 0.253; Figure 2A).

The successful performance of the motion and envelope models was unsurprising given previous work investigating their relationship with EEG (Goncalves et al., 2014; Crosse et al., 2015a). But the fact that a model based on labeling the video with categorical visemic labels was as good at predicting EEG as the others was not trivial and suggests that EEG may be reflecting categorical speech processing. However, it may have been possible that its performance could be attributed to the timing of visual speech onsets irrespective of the particular visemes corresponding to those onsets. We sought to test this by randomizing the particular visemes in the speech stimuli while preserving their onset and offset time points. A significant reduction in performance (*t*_(20)_ = 7.99, *p* = 1.17 × 10^−7^; Figure 2B) demonstrates that timing alone does not account for the performance of the viseme model. Still, further evidence is required to definitively prove that the viseme model indeed captures high-level, linguistic processing of visual speech.

### Complementary Information Provided by Visual Speech Models

In an effort to reveal the encoding of complementary information between the individual models, we looked at the performance of different model combinations. The approach taken here can be explained in view of our understanding of audio speech processing. Phonemes are defined as the smallest unit of audio speech (Chomsky and Halle, 1968), and despite spectro-temporal variations, different occurrences of the same phoneme are categorically perceived (Okada et al., 2010). Similarly, during natural speech, the motion associated with particular visemes varies. This is largely dependent on the location of the viseme in the word, phrase or utterance as well as the speaker and language (Demorest and Bernstein, 1992; Yakel et al., 2000; Soto-Faraco et al., 2007). Thus, the motion model (M) is expected to capture the variation across visemes, while the viseme model (V) categorically labels visually similar phonemes and so is ignorant of these variations. When these models are employed to predict EEG responses to speechreading, it is expected that individually, they should perform similarly, given their complementary strengths, whereas a combination of the two should result in an enhanced representation, thus improving model performance. Therefore, we derived a model based on combining the motion signal with the viseme representation (MV). In line with our hypothesis, we found a significant improvement in performance of this model over the individual models (MV vs. M: *t*_(20)_ = 2.7, *p* = 0.014, MV vs. V: *t*_(20)_ = 6.3, *p* = 3.88 × 10^−6^) suggesting that EEG reflects the processing of both low-level motion fluctuations and higher-level visual speech features (Figure 2A).

In natural speech, it is known that bodily movements do not function independently of lip movements (Yehia et al., 2002; Munhall et al., 2004) and given the reported correlation between lip movements and the acoustic envelope (Chandrasekaran et al., 2009), there exists a degree of redundancy between the motion (M) and envelope (E) models. Nonetheless, during pauses and silent periods we would expect the envelope model to provide a more accurate representation of visual speech (i.e., is zero) than the motion, since any motion at these times is unrelated to speech. However, during speech, we might expect the motion model to be more representative of the visual speech content, since it captures the full range of dynamic visual input present. Thus, combining the envelope and motion models (EM) should result in an improved prediction. As expected we found that EM has an improved prediction accuracy (E: *t*_(20)_ = 6.19, *p* = 4.83 × 10^−6^, M: *t*_(20)_ = 5.03, *p* = 6.37 × 10^−5^; Figure 2A), demonstrating that these models track complementary neural processes in visual regions.

As previously mentioned, the acoustic envelope represents a proxy measure of lip movements. Another approach to representing articulatory movements is according to the categorical speech units with which the lip movements are associated. Whereas the envelope model (E) tracks differences in lip movements for each particular utterance, the viseme model (V) captures their categorical nature. Based on this reasoning, we expect that these models represent distinct stages of visual speech perception and seek to quantify this. Thus, we formed a combined model (EV) and assessed its ability to predict EEG. And while E and V perform similarly, EV has an improved performance over both individual models (E: *t*_(20)_ = 2.42, *p* = 0.025, V: *t*_(20)_ = 3.69, *p* = 0.001; Figure 2A). Although the envelope model (E) represents a good correlate of lip movements, we might well expect the motion model (M) to more comprehensively represent the low-level speech content since it is a more direct measure and captures the full range of motion present, e.g., head, eye movements etc. This is supported by the finding that MV outperforms EV (*t*_(20)_ = 2.23, *p* = 0.037; Figure 2A) suggesting that the motion model may capture more of the low-level visual speech features than those that are captured by the envelope.

To continue with our reasoning that the E, M and V models all capture complementary information about visual speech (Figure 2C), we used a model involving the combination of all three representations. This combined model, EMV, outperforms each of the individual models (E: *t*_(20)_ = 3.95, *p* = 7.90 × 10^−4^, M: *t*_(20)_ = 3.83, *p* = 0.001, V: *t*_(20)_ = 8.17, *p* = 8.40 × 10^−8^). The combined EMV model also outperforms EV (*t*_(20)_ = 6.04, *p* = 6.68 × 10^−6^) and MV (*t*_(20)_ = 3.26, *p* = 0.004), although, despite having the highest mean prediction accuracy, it was not significantly better than EM (*t*_(20)_ = 0.52, *p* = 0.610). This was somewhat surprising, especially given that there was no significant difference in performance between EM and either EV (*t*_(20)_ = 1.93, *p* = 0.069) or MV (*t*_(20)_ = 0.54, *p* = 0.596; Figure 2A).

Finally, we wished to investigate whether or not our cross validation approach had successfully insured us against the risk of improved performance coming about simply as a result of having more free parameters. We did this by comparing the envelope and viseme model (EV; 13 free parameters) with the motion model (M; 1 free parameter) and found no significant difference in performance (*t*_(20)_ = 1.15, *p* = 0.263; Figure 2A). This suggests that generation of a higher dimensional model is not guaranteed to give a significantly improved performance over lower dimensional models. In fact, it is possible that higher dimensional models (i.e., V, and any combination including V) would underperform due to the requirement for greater amounts of data to ensure the models are optimally fit.

### Spatiotemporal Representation of Visual Speech

**Figure 3:**
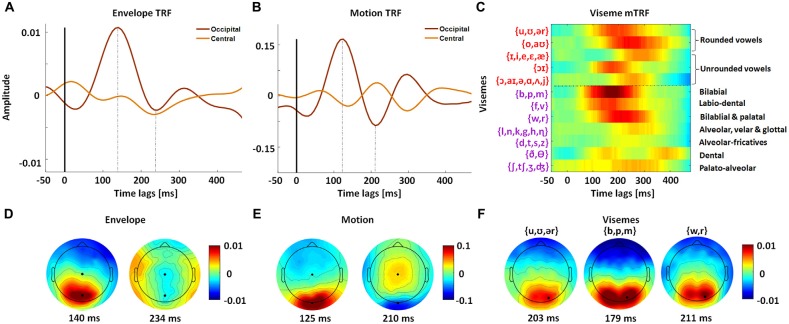
**Spatiotemporal analysis of mTRF models for natural visual speech.** mTRFs plotted for envelope **(A)**, motion **(B)** and visemes **(C)** at peri-stimulus time lags from −50 ms to 500 ms, for representative central and occipital electrode channels. The dotted line separates vowels and consonant visemes. The phonemes contained within each viseme category are shown on the left. **(D)** Topographies of P1 (140 ms) and N1 (234 ms) TRF components for the envelope model. **(E)** Topographies of P1 (125 ms) and N1 (210 ms) TRF components for the motion model. **(F)** Topographies for representative vowel and consonant visemes corresponding to their P1 time points. All visemes have a similar distribution of scalp activity. The black markers in the topographies **(D–F)** indicate the channels for which the corresponding TRFs **(A–C)** were plotted. The viseme mTRF and the topography colorbar are the same.

Since the frame-to-frame motion is precisely time-locked to the stimulus, its TRF has sharp positive and negative components (Figure 3B). In contrast with this, the envelope and viseme TRFs suffer from some smearing effects since the stimuli are aligned with the unheard acoustic signal and so have a complex temporal relationship with the visual input. As a result, they are not quite as precisely time-locked to the EEG (Figures 3A,C). Unsurprisingly, we found that the TRF amplitudes were largest over occipital scalp, suggesting that visual speech is preferentially processed in visual cortex (Figures 3D–F).

The viseme TRF also fits expectations in that visemes associated with clear and extended visibility are more strongly represented in the model weights, e.g., bilabials (/b/, /p/, /m/) and labiodentals (/f/, /v/). The response to these frontal consonants is also much sharper than for the other categories and is consistent with behavioral (Lidestam and Beskow, 2006) and electrophysiological studies (van Wassenhove et al., 2005) of viseme identification. The topographic distribution of these TRF weights also showed markedly different patterns for different classes of visemes (Figure 3F). However we must express caution when examining the mTRF since viseme occurrences are not equal across categories (for all trials: v1 = 4446, v2 = 3274, v3 = 11,197, v4 = 301, v5 = 16,552, v6 = 7416, v7 = 3722, v8 = 16,147, v9 = 20,092, v10 = 6789, v11 = 2421 and v12 = 2992). Furthermore, the delay between viseme onset and phoneme onset varies depending on their location within a particular utterance (e.g., start of word vs. middle of word). Given that our viseme timings were based on a transformation from phoneme timings, this variation may result in suppression as well as smearing of the TRF amplitudes, e.g., alveolar-fricatives (/d/, /t/, /s/, /z/).

## Discussion

In this work, we introduce a framework for investigating the cortical representation of natural visual speech. Specifically, we model how well low- and high-level representations of visual speech are reflected in EEG activity arising from visual cortex. Our results suggest that visual regions are involved in processing the physical stimulus dynamics as well as categorical visual speech features.

### Visual Cortical Entrainment to Envelope, Motion and Visemes Indices during Lipreading

Neural entrainment to continuous visual speech has been previously studied in the context of physical, low-level information through the use of the acoustic envelope (Crosse et al., 2015a) and lip movements (Park et al., 2016). Park et al. (2016) showed that high-level visual regions as well as speech processing regions specifically entrained to the visual component of speech. This is consistent with theories of visual speech encoding through visual pathways (Bernstein and Liebenthal, 2014). Here, we build on these findings to incorporate evidence from perceptual studies (Woodward and Barber, 1960; Fisher, 1968; Auer and Bernstein, 1997), which have identified a basic unit of visual speech, to demonstrate that visually similar phonemes (i.e., visemes) are reflected in low-frequency EEG recordings among persons with normal hearing during lipreading.

Specifically, we provide objective evidence that these features present complementary information to the physical motion present (Figure 2A). Central to this, is the meaningful interpretation of visemes, demonstrated by the significant reduction in performance upon randomization of visemes within their active time points (Figure 2B). This is in line with the idea that a combination of bottom-up (extracting information from the visual speech signal, i.e., motion) and top-down processing (e.g., use of working memory and categorical perception) are involved in visual speech perception (Lidestam and Beskow, 2006). In keeping with evidence that high-level visual speech is processed in visual cortical regions, the observed visemic entrainment was strongest over occipital electrodes (Figures 3D–F). These results also align well with recent work modeling the hierarchical processing of acoustic speech in auditory cortex using analogous techniques (Di Liberto et al., 2015), suggesting that visual cortex may indeed process visual speech in a similar hierarchical fashion (Bernstein and Liebenthal, 2014). In addition, this framework facilitates a more detailed analysis of this notion of hierarchical processing of visual speech through analysis of the timing and distribution of the TRF weights across visemes (Di Liberto et al., 2015). This allows one to examine the sensitivity of neural responses to different viseme categories as a function of response latency. Thus, for a hierarchical processing system, one would expect to see differences in viseme encoding according to the different articulatory features that produced them, and for these differences to be more pronounced at longer latencies. However, due to the method used for generating the viseme model (i.e., based on acoustic timings) it was not appropriate, in this case, to carry out further analysis into the viseme mTRF latencies. It would also be informative to compare our TRFs with ERP research on responses to different phonemes and syllables presented visually. For example, similar to our findings, previous ERP work has shown strong responses to labial consonants (e.g., van Wassenhove et al., 2005; Bernstein et al., 2008; Arnal et al., 2009). One caveat here though is that directly comparing the TRF to ERPs is complicated by the fact that the TRFs are inherently different in terms of how they are derived (see Lalor et al., 2009). Furthermore, our stimuli involve natural speech and so have important differences from repeated presentations of phonemes/syllables (e.g., coarticulation effects, rhythmic properties, complex statistical structure, etc.).

Although the idea of visually indistinguishable phonemes was first described over 50 years ago (Woodward and Barber, 1960), there remains disagreement about how, and to what extent, these are actually perceived. Finding categorical responses to visemes using electrophysiology would be an objective way to provide evidence for high-level neural computations involving visual speech perception. However, the viseme model can also be thought of as shorthand labeling of the detailed motion associated with each viseme. In addition, the envelope and motion regressors surely do not capture all of the detailed, relevant motion patterns associated with these visemes. Hence, the viseme model may simply perform well because it leads to an improved measure of the motion in the video, rather than being a result of higher-level, categorical processing. However, this is unlikely given the finding that EM and EMV perform similarly. Indeed this finding raises the question as to whether or not the information represented by the viseme model is already captured by the combination of the envelope and motion. However, it is evident from the performances of the individual models across subjects that V does not correlate with E or M (Figure 2C), suggesting that it reflects a distinct process in the neural activity. Furthermore, since EMV contains 12 *additional* parameters to EM, it will require more data to ensure the model is optimally fit. This could be remedied by the collection of more data or the development of a generic model for predicting subject’s EEG activity (Di Liberto and Lalor, 2016). Another way to resolve this issue would be to examine the cortical responses to time-reversed visual speech i.e., the frames presented in reverse order, which would facilitate the isolation of motion responses from speech specific responses. In fact, time-reversed visual speech contains segments that are not different from forward speech, such as vowels and transitions into and out of consonants (Ronquest et al., 2010; Bernstein and Liebenthal, 2014). It would also maintain similar low-level information, such as variation in motion between frames and rhythmic pattern. However it would no longer be identified as speech due to removal of lexical information (Paulesu et al., 2003; Ronquest et al., 2010), thus depleting any speech-specific processing effects seen in the forward speech models. In this case, we would expect the performance of the motion model to remain similar, while the visemes performance should be significantly reduced.

Our finding that prediction accuracy of EEG activity using the acoustic envelope is similar to that of motion and visemes (Figure 2A) is in line with work showing that occipital channels best reflect the dynamics of the acoustic envelope during lipreading (Crosse et al., 2015b). This may not reflect entrainment to the speech envelope *per se*, but perhaps to speech related movements which are highly correlated with the envelope (Chandrasekaran et al., 2009). Thus, the improved performance demonstrated by the combination of the envelope with the motion model may be explained by reports that visual cortex processes localized and global motion from a natural scene at specialized regions (Bartels et al., 2008). However, our finding that MV outperforms EV suggests there is a greater amount of mutual information between the envelope and viseme models and this may be explained by an analysis-by-synthesis perspective of visual speech encoding. Such a mechanism has previously been implicated to underpin envelope tracking in auditory cortex (Ding et al., 2013) and may also be responsible for the observed entrainment in visual regions, reflecting an internal synthesis of visual speech features. Work from van Wassenhove et al. (2005) led to a proposal whereby an analysis-by-synthesis mechanism involves perceptual categorization of visual inputs which are used to evaluate auditory inputs. This mechanism has also been suggested by Crosse et al. (2015a), following the finding of a strong correlation between behavior and envelope tracking during lipreading, and is in line with results presented here. In contrast with this, work from Park et al. (2016) did not find the acoustic envelope to be coherent with MEG activity in visual cortex. This could be explained by the intrinsic difference between MEG and EEG recordings, where MEG measures current flow tangential to the scalp whereas EEG is sensitive to both tangential and radial components. An alternative explanation is due to differences in study design, since their task did not require subjects to concentrate on lipreading. Instead, subjects attended to audio speech whereby the visual speech was either informative (i.e., matched the attended audio speech) or distracting (i.e., unmatched).

The combined models used here provide us with a means to quantify the differential tracking of particular stimulus features in the EEG. However, the contribution from different stimuli to the EEG prediction could be more clearly defined by regressing out the common variability between the predictors, thus creating independent predictors. One way to achieve this is using partial coherence, which removes the linear contribution of one predictor (e.g., the motion signal) from another (e.g., visemes) in order to reveal entrainment to visemes which cannot be accounted for by the motion signal. This approach has been used previously to separate neural entrainment to lip movements from the speech envelope (Park et al., 2016). Applying this method within the framework presented here could shed light on the unique variability captured by each stimulus in the EEG, and coupled with a high spatial resolution imaging technique, such as fMRI, one could also localize these entrained regions.

### Limitations and Future Directions

It is important to consider some limitations of the current work. First, this experiment was not specifically designed as a visual-only speech experiment and so there are a couple of considerations with regard to how this particular paradigm may have influenced results seen here. In the original experiment, there were seven conditions, six of which contained audio speech. Thus, subjects may have become familiar with the audio content before viewing the visual speech condition. However, to minimize the effect of memory, presentation order of the 105 trials (15 stimuli × 7 speech conditions) was completely randomized within participants. And if subjects were able to relate the silent speech back to a previously heard audio trial, then we would expect to see a much improved target word detection. However, the consistently poor target word detection scores (36.8 ± 18.1%, Crosse et al., 2015a) suggests that subjects were not good at recognizing the speech from an earlier condition. Another issue is that the use of a well-known speaker may have aided subjects’ lipreading ability. However, in the present experiment, the subjects were not familiar with the content of the speech and, as mentioned above, their lipreading performance was relatively poor. Therefore, it is reasonable to assume that these factors did not have a major influence on the results seen here. Another issue with using a well-known speaker is that subjects may have strongly imagined the audio speech that accompanied our silent videos. If so, one might expect to see evidence of auditory cortical tracking of such imagined audition. Indeed, we have previously sought to uncover EEG evidence for such a phenomenon with well-known speech (Crosse et al., 2015b). However, the evidence we have found for this has been weak at best. And, again, because in the present experiment the content was unfamiliar, we do not expect imagined audition will have played a significant role.

One other limitation of the present experiment is that the viseme representation of the speech used here is sub-optimal since the viseme stimulus is derived from a phoneme alignment of the speech signal before transformation into viseme groups. The accuracy of this alignment can be affected by different representations of the same phonemes. For example, we might expect that the alignment work better for the /b/ phoneme since this is associated with a large spike in the spectrogram and so is easier for the software to identify. This can in turn result in a more effective mapping of stimulus to EEG compared with phonemes which may be more difficult to accurately align (e.g., /s/ or /z/). Secondly, since it is essentially a phonetic mapping, the visemes are tied to the acoustic boundaries, and given the complex temporal relationship between audio and visual speech the viseme timings are imprecise. Thus, when these features are mapped to EEG, these slight variations can cause smearing of response peaks (as seen in Figure 3C). It is also important to keep in mind the poor lipreading ability of normal hearing subjects. We would expect to see a large boost in viseme model performance for trained lipreaders or subjects familiar with the speech content due to an improved ability to recognize the silent speech (Bernstein et al., 2000). Nevertheless, our results are consistent with the notion that the visual system interprets visual speech and takes a first step to investigate how the visual system may represent the rich psycholinguistic structure of visual speech.

## Conclusion

In summary, we have presented a framework to objectively assess the possibility of speech-sensitive regions in visual cortex encoding high-level, categorical speech features. The results presented are akin to findings in the auditory domain and support the theory that visual regions are involved in categorical speech perception (Bernstein and Liebenthal, 2014). Future work will seek to strengthen the evidence provided here, for example, by applying these models to subjects watching known vs. unknown speech, or by recruiting individuals with hearing impairments who have superior speechreading ability. In addition, the coupling of these models with recently developed representations of auditory speech (Di Liberto et al., 2015) may shed light on how the brain weights sensory inputs from different modalities to form a multi-sensory percept.

## Author Contributions

AEO and MJC contributed equally to the work. MJC and ECL designed research; MJC collected data; AEO performed research; AEO, MJC and GMDL analyzed data; AEO, MJC, GMDL and ECL interpreted data and wrote the article.

## Funding

This work was supported by the Programme for Research in Third-Level Institutions and co-funded under the European Regional Development fund. Additional support was provided by the Irish Research Council’s Government of Ireland Postgraduate Scholarship Scheme.

## Conflict of Interest Statement

The authors declare that the research was conducted in the absence of any commercial or financial relationships that could be construed as a potential conflict of interest.
